# Optimization of Covert Communication in Multi-Sensor Asymmetric Noise Systems

**DOI:** 10.3390/s24030796

**Published:** 2024-01-25

**Authors:** Sen Qiao, Ruizhi Zhu, Xiaopeng Ji, Junjie Zhao, Huihui Ding

**Affiliations:** School of Electronics and Information Engineering, Nanjing University of Information Science and Technology, Nanjing 210044, Chinadinghuihui@nuist.edu.cn (H.D.)

**Keywords:** covert communication, asymmetric noise, BPSK, Taylor series expansion

## Abstract

This work investigates wireless covert communication in a multi-sensor asymmetric noise scenario. We adopt KL (Kullback–Leibler) divergence as the covertness constraint metric and mutual information as the transmission rate metric. To accurately approximate KL divergence and mutual information in covert communication, we employ the Taylor series expansion technique. Analytical expressions for KL divergence and mutual information in covert communication are derived, and we optimize the amplitude gain and phase angles based on these analytical expressions. Our findings underscore the importance of phase angle selection in covert communication within asymmetric noise systems. We propose an effective method for optimizing the transmission amplitude gain and phase angles in scenarios with asymmetric noise. Numerical results validate the effectiveness and superiority of our proposed method.

## 1. Introduction

With the rapid development of Internet of Things (IoT) technology, the demand for information transmission from sensors is also growing exponentially [1,2]. Meanwhile, the security issues related to information transmission in the Internet of Things (IoT) will become one of the primary tasks in the future [3,4,5]. Traditional encryption methods and physical layer security techniques aim to prevent eavesdropping on communication content [6,7], but merely protecting communication content is no longer sufficient to meet the current requirements for information security [8]. Even if the information is encrypted, metadata such as network traffic patterns may still leak some sensitive information [9].

In order to hide communication behaviors, covert communication technology has been proposed [10]. In battlefield environments or confrontational areas, even a little exposed intention of communication may lead to significant strategic failures [11]. Consequently, the military has developed diverse techniques (e.g., the spread spectrum technique [8,12,13]) to ensure the covertness of communication, i.e., to hide the presence of communication from watchful adversaries. Although numerous covert communication methods have been proposed, such as encoding information on top of the training sequences of WiFi [14], the cyclic prefix of WiFi OFDM symbols [15], or a dirty WiFi QPSK constellation [16], the theoretical boundary of covert communication was not investigated until *square root law* (SRL) was discovered for additive white Gaussian noise (AWGN) channels in [17].

In covert communication scenarios, the transmitter, Alice, aims to transmit messages to the legitimate receiver, Bob, through a noisy channel while ensuring detection evasion by an adversary, Willie [18]. While some previous works have investigated covert communication in IoT scenarios, such as [2,19,20,21], these works specifically concentrate on scenarios characterized by symmetric noise. In these scenarios, the variances of the real and imaginary axes of complex Gaussian noise in the channel are equal.

In scenarios with a large number of sensor devices, the uneven distribution and uncertain transmission times of these devices lead to unequal noise levels in different directions, resulting in asymmetric noise scenarios. Due to the asymmetric noise interference caused by the distribution and operational states of sensors, it is necessary to investigate covert communication in asymmetric noise scenarios. In many cases, some complex coding techniques such as Polar codes [22,23] and LDPC codes [24,25] can address noise-related challenges. However, coding introduces additional communication overhead (i.e., Alice) and imposes higher hardware requirements on the decoding end (i.e., Bob). Therefore, we aim to investigate some technologies on the physical layer of covert communication, aiming to identify low-cost methods that enhance the reliability of covert communication.

**Figure 1:**
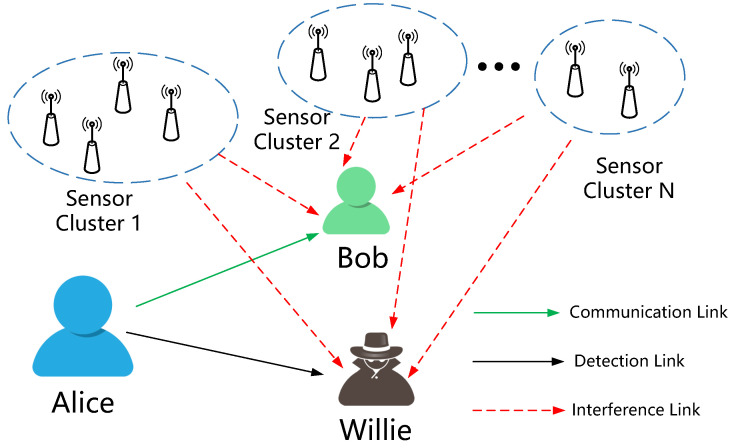
Covert communication in multi-sensor systems.

In this work, we investigate wireless covert communication in a multi-sensor asymmetric noise scenario. As shown in Figure 1, our communication scenario includes the covert information transmitter Alice, covert information receiver Bob, covert communication detector Willie, and *N* randomly distributed sensor clusters. In our scenario, *N* randomly distributed sensor clusters are unevenly distributed around Alice, Bob, and Willie, and each sensor cluster transmits signals with different probabilities and periods. To enhance the alignment of theoretical outcomes with practical communication scenarios, we employ a Binary Phase Shift Keying (BPSK) codebook, instead of a Gaussian codebook. It is worth noting that, in order to maximize the transmission rate under the same covert constraints, Alice can optimize the amplitude gain and initial phase angle of the transmitted signal to enhance the performance of covert communication. The main contributions of this paper are summarized as follows:

(1) We firstly investigate the theoretical boundaries of covert communication in asymmetric noise scenarios. Leveraging Taylor series expansion, we provided accurate approximations for the reliability and covert properties in these scenarios.
(2) We proposed a physical layer optimization approach for covert communication in asymmetric noise systems, optimizing transmission amplitude and initial phase angle. This method effectively improves the covert channel capacity without complex encoding and decoding operations.
(3) Building upon Taylor approximations using KL divergence and mutual information, we refined the optimization method. Through mathematical transformations, the multi-parameter optimization problem degenerates into an initial angle optimization problem, greatly reducing the complexity of the optimization approach.
(4) Based on our theoretical framework, we conducted simulation validations of covert communication in asymmetric noise scenarios. Experimental results confirmed our proposed theory, demonstrating that maximizing covert communication channel capacity can be achieved through simple angle selection.

The remainder of this paper is organized as follows. Section 2 introduces related works. In Section 3, the modeling of asymmetric noise communication scenarios is introduced, encompassing a scenario overview and problem formalization. In Section 4, the performance analysis of covert communication is provided, including transmission rates and covertness performance. In Section 5, the optimization method of transmission amplitude and phase angles is proposed. Section 6 presents numerical simulations to evaluate the outcomes of the optimization. Finally, Section 7 provides the conclusion and summary of the paper.

## 2. Related Works

In existing covert communication theory research, theoretical boundaries and performance achievability have been extensively explored over real Gaussian channels or noise symmetry channels. In [10], Bash et al. proposed SRL, which states that to ensure both covertness and reliability, only $O\left(\sqrt{n}\right)$ bits can be transmitted over *n* channel uses. Note that the transmission rate approaches zero as *n* grows to infinity. This seminal theorem has subsequently been extended to various channel models, including discrete memoryless channels [26,27,28], binary symmetric channels [29], state-dependent channels [30], and multi-user channels [31,32,33,34,35], etc.

Many studies in covert communication theory have been dedicated to the analysis of theoretical boundaries, focusing on covert communication achievability over real Gaussian channels. In [36], the authors employed a slotted AWGN channel model with T(n) slots each containing *n* symbol periods, where Alice may use a single slot out of T(n). They proved that in the scenario of a real Gaussian channel, by randomly selecting a single slot, $O\left(\min\{\sqrt{n\log T(n)},n\}\right)$ bits can be transmitted over *n* channel uses. In [30], the authors investigate covert communication over a state-dependent real Gaussian channel, where Alice has causal or noncausal knowledge of the channel states, and derive closed-form formulas for the maximum achievable covert communication rate for discrete memoryless channels. In [37], Sobers et al. investigated the covert communication in complex Gaussian channels, where the Gaussian noise is symmetrical, and they proved that with the assistance of a jammer, Alice can transmit covert information at a positive rate. In [12], the authors investigate covert ambient backscatter communications in complex Gaussian channels, and their analyses show that the covert transmission rate is subject to the SRL. Then, the phase angle gain over complex Gaussian channels was discovered in [11], and we can obtain a phase gain as $\sqrt{2}$ with a simple operation. Moreover, the theoretical boundary of covert communication in backscatter systems has been further explored in [38], and additional covert gain can be obtained from signals reflected without decoding.

While some studies have investigated covert communication in complex Gaussian channels, existing works have primarily focused on scenarios where the noise in the real and imaginary axes is symmetric. Moreover, in [11], the authors demonstrated that the initial phase angle of BPSK codewords does not affect the covert nature and transmission rate in noise symmetry scenarios. In this work, we aim to explore whether, in asymmetric noise scenarios, the initial phase angle affects the transmission rate and covertness performance. Additionally, we attempt to optimize the amplitude gain and initial phase angle for covert communication in an asymmetric noise scenario.

## 3. System Model

### 3.1. Communication Scenario

As illustrated in Figure 1, we consider a wireless covert communication scenario involving multiple sensor clusters, including the covert communication transmitter Alice, covert communication receiver Bob, covert communication detector Willie, and multiple sensor clusters. Alice, Bob, and Willie are equipped with a single antenna. Multiple sensor clusters transmit signals with different Gaussian codewords, and Alice, Bob, and Willie are unable to decode and eliminate interference from the signals of multiple sensors. We consider a quasi-static block fading model where the channel is static and frequency-flat within each coherent interval containing *n* symbols. The signal received by Bob and Willie can be expressed as

(1) $$\begin{array}{cc} {\hat{Z}}_{b} & =h_{b}D+\sum_{q=1}^{N}h_{q,b}s_{q}+{\hat{n}}_{b}, \end{array}$$

(2) $$\begin{array}{cc} {\hat{Z}}_{w} & =h_{w}D+\sum_{q=1}^{N}h_{q,w}s_{q}+{\hat{n}}_{w}, \end{array}$$

respectively, where D ∈C is the symbol transmitted by Alice, $s_{q}$ is the symbol sent by the *q* sensor cluster, and $h_{b}\in C$, $h_{w}\in C$, $h_{q,b}\in C$ and $h_{q,w}\in C$ are Alice-to-Bob and Alice-to-Willie, the *q*-th sensor cluster to Bob, the *q*-th sensor cluster to Willie channel coefficient, which are assumed to be acceptable to everyone. Furthermore, ${\hat{n}}_{b}\in C$ and ${\hat{n}}_{w}\in C$ denote the zero-mean complex-valued Gaussian noise vectors with the covariance $2{\hat{\sigma }}_{b}^{2}$ and $2{\hat{\sigma }}_{w}^{2}$. Assuming that all *N* sensor clusters transmit information with a Gaussian signal, the power of the received signal at Bob is $P_{N,b}≜E\{|\sum_{q=1}^{N}h_{q,b}s_{q}{|}^{2}\}$, and the power of the received signal at Willie is given by $P_{N,w}≜E\{|\sum_{q=1}^{N}h_{q,w}s_{q}{|}^{2}\}$. It is worth noting that, instead of adopting the conventional assumption of Gaussian input data, we let D be the symbol randomly selected from well-known BPSK constellations set with amplitude gain β, i.e., {−β,+β}. Considering the signal composition of the sensors, we can equivalently rewrite the channel as

(3) $$\begin{array}{cc} Z_{b} & =h_{b}D+n_{b}, \end{array}$$

(4) $$\begin{array}{cc} Z_{w} & =h_{w}D+n_{w}, \end{array}$$

where $n_{b}∼\mathrm{CN}\left(0,2\sigma_{b}^{2}\right)$ and $n_{w}∼\mathrm{CN}\left(0,2\sigma_{w}^{2}\right)$ denote the equivalent noise after superimposing the sensor signals, with $\sigma_{b}^{2}=\frac{2{\hat{\sigma }}_{b}^{2}+P_{N,b}}{2}$ and $\sigma_{w}^{2}=\frac{2{\hat{\sigma }}_{w}^{2}+P_{N,w}}{2}$. In the following context, we will analyze and optimize our problem based on equivalent channels (3) and (4).

### 3.2. Transmission Scheme

By employing random coding generation, Alice encodes a message *M* into a codeword $D^{n}=\left[D_{1},...,D_{n}\right]\in C^{n}$. For the *i*th symbol, its amplitude is β and its phase angle is independently selected from {θ+π,θ} with equal probability [11,39]. Next, we define *B* and $\Delta_{b}$ as the amplitude coefficient and the phase of complex value $h_{b}$. The codeword $D^{n}$ is generated independently and identically distributed (i.i.d.) according to the following probability distribution:

(5) $$\begin{array}{c} P\left(D^{n}\right)=\prod_{i=1}^{n}P_{D}\left(D_{i}\right). \end{array}$$

Bob observes $Z_{b}^{n}=\left[Z_{b,1},\ldots ,Z_{b,n}\right]\in C^{K\times n}$ over the wireless channel and decodes the covert messages with his knowledge. The transmission rate is measured by the mutual information between the discrete input $D^{n}$ and the channel output $Z_{b}^{n}$, which is given by

(6) $$\begin{array}{cc} I(Z_{b}^{n};D^{n}) & =I(Z_{b,1},\ldots ,Z_{b,n};D_{1},\ldots ,D_{n}). \end{array}$$

Bob possesses knowledge regarding the construction of the codebook, including the amplitude gain β and phase angles {θ+π,θ}, and he receives the symbols corrupted by AWGN. Then, we give the following joint distribution of $Z_{b}^{n}$ and $D^{n}$ as follows:

(7) $$\begin{array}{c} P\left(Z_{b}^{n}D^{n}\right)=P_{D}^{n}\left(D^{n}\right)\times W_{Z_{b}^{n}|D^{n}}\left(Z_{b}^{n}|D^{n}\right). \end{array}$$

The channel probability transition matrix $W_{Z_{b}^{n}{|\left(D\right)}^{n}}$ is given by

(8) $$\begin{array}{c} W_{Z_{b}^{n}|D^{n}}=\prod_{i=1}^{n}W_{Z_{b,i}|D_{i}}, \end{array}$$

with

(9) $$\begin{array}{cc} \; & W_{Z_{b,i}|D_{i}}\left(Z_{b,i}=\left(x,y\right)|D_{i}=\left(\beta \cos\theta ,\beta \sin\theta \right)\right)\\ \; & =\frac{1}{2\pi \sqrt{\sigma_{b,x}\sigma_{b,y}}}\exp\left(-\frac{{\left(x+B\beta \cos(\Delta_{b}+\theta )\right)}^{2}}{2\sigma_{b,x}^{2}}-\frac{{\left(y+B\beta \sin(\Delta_{b}+\theta )\right)}^{2}}{2\sigma_{b,y}^{2}}\right), \end{array}$$

and

(10) $$\begin{array}{cc} \; & W_{Z_{b,i}|D_{i}}\left(Z_{b,i}=\left(x,y\right)|D_{i}=\left(\beta \cos\left(\theta +\pi \right),\beta \sin\left(\theta +\pi \right)\right)\right)\\ \; & =\frac{1}{2\pi \sqrt{\sigma_{b,x}\sigma_{b,y}}}\exp\left(-\frac{{\left(x-B\beta \cos(\Delta_{b}+\theta )\right)}^{2}}{2\sigma_{b,x}^{2}}-\frac{{\left(y-B\beta \sin(\Delta_{b}+\theta )\right)}^{2}}{2\sigma_{b,y}^{2}}\right), \end{array}$$

where $\sigma_{b,x}^{2}$ and $\sigma_{b,y}^{2}$ represent the variances of the complex Gaussian noise at Bob on the *x*-axis and *y*-axis.

### 3.3. Hypothesis Test

Willie conducts a binary hypothesis test [10] based on *n* consecutive observations $Z_{w}^{n}=\left[Z_{w,1},\ldots ,Z_{w,n}\right]\in C^{n}$ to determine whether Alice is communicating to Bob. Let $\sigma_{w,x}^{2}$ and $\sigma_{w,y}^{2}$ denote the variance of the complex Gaussian noise at Willie on the *x*-axis and *y*-axis; *A* and $\Delta_{w}$ denote the amplitude coefficient and phase angle of $h_{w}$. Specifically, the null hypothesis ($H_{0}$) posits the absence of communication, where each sample $Y_{w,i}=n_{w,i}$ is an independent and identically distributed (i.i.d.) complex Gaussian random variable following the distribution CN(0, $\sigma_{w}^{2}$) with $\sigma_{w}^{2}=\sigma_{w,x}^{2}+\sigma_{w,y}^{2}$. On the other hand, the alternative hypothesis ($H_{1}$) suggests communication is occurring, and each sample $Y_{w,i}=h_{w}D_{i}+n_{w,i}$. Willie’s objective is to discriminate between these two hypotheses:

(11)H0:Zw=nw,(12)H1:Zw=hwD+nw,

where $h_{w}$ is the channel coefficient from Alice to Willie. Let $Q_{1}^{n}$ (resp. $Q_{0}^{n}$) represent the input distribution corresponding to Willie’s *n* observations under the conditions of $H_{1}$ (resp. $H_{0}$), respectively. The probability of a *false alarm*, rejecting $H_{0}$ when it is true, is denoted by $P_{FA}$, while the probability of *missed detection*, accepting $H_{0}$ when it is false, is denoted by $P_{MD}$.

Willie has knowledge of the distributions $Q_{1}^{n}$ and $Q_{0}^{n}$ and can conduct an optimal statistical hypothesis test such that

(13) $$\begin{array}{c} P_{FA}+P_{MD}\geq 1-\sqrt{D(Q_{1}^{n}∥Q_{0}^{n})}, \end{array}$$

where $D(Q_{1}^{n}∥Q_{0}^{n})$ represents the KL divergence between $Q_{1}^{n}$ and $Q_{0}^{n}$. The objective is to achieve covert communication by ensuring that the sum of error probabilities is one, i.e., $P_{FA}+P_{MD}=1$. This implies making $D(Q_{1}^{n}∥Q_{0}^{n})$ negligible [18,40,41]. Specifically, we can ensure the covertness of communication by guaranteeing

(14) $$\begin{array}{c} D(Q_{1}^{n}∥Q_{0}^{n})\leq \epsilon , \end{array}$$

where ϵ is an arbitrarily small value within the range (0, 1).

Willie possesses knowledge regarding the construction of the codebook and channel coefficient from Alice to Willie, and he receives the symbols corrupted by AWGN. The distribution of $Q_{0}$ and $Q_{1}$ can be expressed as

(15) $$\begin{array}{cc} Q_{0}\left(x,y\right) & =\frac{1}{2\pi \sigma_{w,x}\sigma_{w,y}}\exp\left(-\frac{x^{2}}{2\sigma_{w,x}^{2}}-\frac{y^{2}}{2\sigma_{w,y}^{2}}\right),\\ Q_{1}\left(x,y\right) & =\frac{1}{2}\frac{1}{2\pi \sigma_{w,x}\sigma_{w,y}}[\exp\left(-\frac{{\left(x-A\beta \cos\left(\Delta_{w}+\theta \right)\right)}^{2}}{2\sigma_{w,x}^{2}}-\frac{{\left(y-A\beta \sin\left(\Delta_{w}+\theta \right)\right)}^{2}}{2\sigma_{w,y}^{2}}\right) \end{array}$$

(16) $$\begin{array}{cc} \; & +\exp\left(-\frac{{\left(x+A\beta \cos\left(\Delta_{w}+\theta \right)\right)}^{2}}{2\sigma_{w,x}^{2}}-\frac{{\left(y+A\beta \sin\left(\Delta_{w}+\theta \right)\right)}^{2}}{2\sigma_{w,y}^{2}}\right)], \end{array}$$

where $\sigma_{w,x}^{2}$ and $\sigma_{w,y}^{2}$ represent the variances of the complex Gaussian noise at Willie on the *x*-axis and *y*-axis.

### 3.4. Problem Formulation

In this work, we aim to investigate the transmission design with the goal of maximizing the mutual information in (6) while satisfying the covertness constraint (14). Our emphasis is on optimizing the amplitude gain β and the phase angle θ between the *x*-axis and *y*-axis. The problem of covert communication in asymmetric noise systems is formulated as

(17)P1:maxβ,θI(Zbn;Dn)(18)s.t.D(Q1n∥Q0n)≤ϵ,(19)0<β,(20)0<θ<π.

## 4. Performance Analysis of the Covert Transmission

### 4.1. Analysis of Transmission Performance

The mutual information can be expressed as

(21) $$\begin{array}{c} I\left(Z_{b}^{n};D^{n}\right)=nI\left(Z_{b};D\right). \end{array}$$

Then, we have

(22)I(Zb;D)=H(Zb)−H(Zb|D)(23)=−∫∫pb(x,y)logpb(x,y)dxdy+∫∫∑t=1212pb,t(x,y)logpb,t(x,y)dxdy(24)=−∫∫∑s=1212pb,s(x,y)log∑t=1212pb,t(x,y)dxdy+∫∫∑s=1212pb,s(x,y)logpb,s(x,y)dxdy(25)=−∫∫∑s=1212pb,s(x,y)log∑t=1212pb,t(x,y)pb,s(x,y)dxdy,

where

(26) $$\begin{array}{cc} p_{b,1}\left(x,y\right) & =\frac{1}{2\pi \sigma_{b}^{2}}\left[\exp\left(-\frac{{\left(x+B\beta \cos(\Delta_{b}+\theta )\right)}^{2}}{2\sigma_{b}^{2}}-\frac{{\left(y+B\beta \sin(\Delta_{b}+\theta )\right)}^{2}}{2\sigma_{b}^{2}}\right)\right], \end{array}$$

(27) $$\begin{array}{cc} p_{b,2}\left(x,y\right) & =\frac{1}{2\pi \sigma_{b}^{2}}\left[\exp\left(-\frac{{\left(x-B\beta \cos(\Delta_{b}+\theta )\right)}^{2}}{2\sigma_{b}^{2}}-\frac{{\left(y-B\beta \sin(\Delta_{b}+\theta )\right)}^{2}}{2\sigma_{b}^{2}}\right)\right]. \end{array}$$

Performing Taylor expansion, we have

(28) $$\begin{array}{cc} \; & \log\left[\frac{\sum_{i=1}^{2}\frac{1}{2}p_{b,i}\left(x,y\right)}{p_{b,1}\left(x,y\right)}\right]=\beta \left(\frac{B\cos(\Delta_{b}+\theta )x}{\sigma_{b,x}^{2}}+\frac{B\sin(\Delta_{b}+\theta )y}{\sigma_{b,y}^{2}}\right)\\ \; & +\beta^{2}\left(\frac{B^{2}{\cos(\Delta_{b}+\theta )}^{2}x^{2}}{2\sigma_{b,x}^{4}}+\frac{B^{2}{\sin(\Delta_{b}+\theta )}^{2}y^{2}}{2\sigma_{b,y}^{4}}+\frac{B^{2}\cos(\Delta_{b}+\theta )\sin(\Delta_{b}+\theta )xy}{\sigma_{b,x}^{2}\sigma_{b,y}^{2}}\right)+O\left(\beta^{3}\right), \end{array}$$

and

(29) $$\begin{array}{cc} \; & \log\left[\frac{\sum_{i=1}^{2}\frac{1}{2}p_{b,i}\left(x,y\right)}{p_{b,2}\left(x,y\right)}\right]=-\beta \left(\frac{B\cos(\Delta_{b}+\theta )x}{\sigma_{b,x}^{2}}+\frac{B\sin(\Delta_{b}+\theta )y}{\sigma_{b,y}^{2}}\right)\\ \; & +\beta^{2}\left(\frac{B^{2}{\cos(\Delta_{b}+\theta )}^{2}x^{2}}{2\sigma_{b,x}^{4}}+\frac{B^{2}{\sin(\Delta_{b}+\theta )}^{2}y^{2}}{2\sigma_{b,y}^{4}}+\frac{B^{2}\cos(\Delta_{b}+\theta )\sin(\Delta_{b}+\theta )xy}{\sigma_{b,x}^{2}\sigma_{b,y}^{2}}\right)+O\left(\beta^{3}\right). \end{array}$$

With some calculations, we can obtain

$$\begin{array}{cc} \; & \;\int \int \;\sum_{j=1}^{2}\frac{1}{2}p_{b,j}\left(x,y\right)\log\left[\frac{\sum_{i=1}^{2}\frac{1}{2}p_{b,i}\left(x,y\right)}{p_{b,j}\left(x,y\right)}\right]dxdy\\ \; & =-\left(\frac{B^{2}{\cos(\Delta_{b}+\theta )}^{2}\beta }{\sigma_{b,x}^{2}}+\frac{B^{2}{\sin(\Delta_{b}+\theta )}^{2}\beta }{\sigma_{b,y}^{2}}\right)\beta \end{array}$$

(30) $$\begin{array}{c} \;+\left(\frac{B^{2}{\cos(\Delta_{b}+\theta )}^{2}}{2\sigma_{b,x}^{2}}+\frac{B^{2}{\sin(\Delta_{b}+\theta )}^{2}}{2\sigma_{b,y}^{2}}\right)\beta^{2}+O\left(\beta^{3}\right) \end{array}$$

(31) $$\begin{array}{cc} \; & =-\left(\frac{B^{2}{\cos(\Delta_{b}+\theta )}^{2}}{2\sigma_{b,x}^{2}}+\frac{B^{2}{\sin(\Delta_{b}+\theta )}^{2}}{2\sigma_{b,y}^{2}}\right)\beta^{2}+O\left(\beta^{3}\right). \end{array}$$

Combining with (22)–(31), the mutual information can be expressed as

(32) $$\begin{array}{c} I\left(Z_{b};D\right)=\frac{\beta^{2}}{2}\left(\frac{B^{2}{\cos(\Delta_{b}+\theta )}^{2}}{\sigma_{b,x}^{2}}+\frac{B^{2}{\sin(\Delta_{b}+\theta )}^{2}}{\sigma_{b,y}^{2}}\right). \end{array}$$

### 4.2. Analysis of Covertness Performance

The expression of KL divergence is given by

(33) $$\begin{array}{c} D\left(Q_{1}∥Q_{0}\right)={\;\int \int \;}_{-\infty }^{\infty }Q_{1}\left(x,y\right)\log\frac{Q_{1}\left(x,y\right)}{Q_{0}\left(x,y\right)}dxdy. \end{array}$$

Recalling the distribution of $Q_{0}$ and $Q_{1}$ in (15) and (16), we can obtain

$$\begin{array}{cc} \log\frac{Q_{1}\left(x,y\right)}{Q_{0}\left(x,y\right)} & =\log\frac{1}{2}[\exp(-\frac{A^{2}\beta^{2}\cos{\left(\Delta_{w}+\theta \right)}^{2}-2xA\beta \cos\left(\Delta_{w}+\theta \right)}{2\sigma_{w,x}^{2}}\\ & -\frac{A^{2}\beta^{2}\sin{\left(\Delta_{w}+\theta \right)}^{2}-2y\beta \sin{\left(\Delta_{w}+\theta \right)}^{2}}{2\sigma_{w,y}^{2}})\\ \; & \;+\exp(-\frac{A^{2}\beta^{2}\cos{\left(\Delta_{w}+\theta \right)}^{2}+2xA\beta \cos\left(\Delta_{w}+\theta \right)}{2\sigma_{w,x}^{2}} \end{array}$$

(34) $$\begin{array}{c} -\frac{A^{2}\beta^{2}\sin{\left(\Delta_{w}+\theta \right)}^{2}+2y\beta \sin{\left(\Delta_{w}+\theta \right)}^{2}}{2\sigma_{w,y}^{2}})]. \end{array}$$

Performing Taylor expansion, the Equation (34) can be expressed as

(35) $$\begin{array}{c} \log\frac{Q_{1}\left(x,y\right)}{Q_{0}\left(x,y\right)}=\phi_{1}\beta^{2}+\phi_{2}\beta^{4}+O\left(\beta^{5}\right), \end{array}$$

with

(36) $$\begin{array}{cc} \phi_{1} & =-\frac{A^{2}\cos{\left(\Delta_{w}+\theta \right)}^{2}}{2\sigma_{w,x}^{2}}-\frac{A^{2}\sin{\left(\Delta_{w}+\theta \right)}^{2}}{2\sigma_{w,y}^{2}}\\ & +\frac{A^{2}\cos\left(\Delta_{w}+\theta \right)\sin\left(\Delta_{w}+\theta \right)xy}{\sigma_{w,x}^{2}\sigma_{w,y}^{2}}+\frac{A^{2}\sin{\left(\Delta_{w}+\theta \right)}^{2}y^{2}}{2\sigma_{w,y}^{4}}+\frac{A^{2}\cos{\left(\Delta_{w}+\theta \right)}^{2}x^{2}}{2\sigma_{w,x}^{4}}, \end{array}$$

(37) $$\begin{array}{cc} \phi_{2} & =-\frac{A^{4}\cos{\left(\Delta_{w}+\theta \right)}^{4}x^{4}}{12\sigma_{w,x}^{8}}-\frac{A^{4}\sin{\left(\Delta_{w}+\theta \right)}^{4}y^{4}}{12\sigma_{w,y}^{8}}-\frac{A^{4}\cos{\left(\Delta_{w}+\theta \right)}^{2}\sin{\left(\Delta_{w}+\theta \right)}^{2}}{2\sigma_{w,x}^{4}\sigma_{w,y}^{4}}\\ \; & \;-\frac{A^{4}\cos{\left(\Delta_{w}+\theta \right)}^{3}\sin\left(\Delta_{w}+\theta \right)x^{3}y}{3\sigma_{w,x}^{6}\sigma_{w,y}^{2}}-\frac{A^{4}\cos\left(\Delta_{w}+\theta \right)\sin{\left(\Delta_{w}+\theta \right)}^{3}xy^{3}}{3\sigma_{w,x}^{2}\sigma_{w,y}^{6}}. \end{array}$$

The expression of KL divergence can be expressed as

(38) $$\begin{array}{c} D\left(Q_{1}∥Q_{0}\right)={\;\int \int \;}_{-\infty }^{\infty }Q_{1}\left(x,y\right)\left(\phi_{1}\beta^{2}+\phi_{2}\beta^{4}+O\left(\beta^{5}\right)\right)dxdy. \end{array}$$

With some calculation, we can obtain the first term as

(39) $$\begin{array}{cc} \; & {\;\int \int \;}_{-\infty }^{\infty }Q_{1}\left(x,y\right)\phi_{1}\beta^{2}dxdy\\ & =-\frac{A^{2}\cos{\left(\Delta_{w}+\theta \right)}^{2}\beta^{2}}{2\sigma_{w,x}^{2}}-\frac{A^{2}\sin{\left(\Delta_{w}+\theta \right)}^{2}\beta^{2}}{2\sigma_{w,y}^{2}}+\frac{A^{4}\cos{\left(\Delta_{w}+\theta \right)}^{2}\sin{\left(\Delta_{w}+\theta \right)}^{2}\beta^{4}}{\sigma_{w,x}^{2}\sigma_{w,y}^{2}}\\ \; & +\frac{A^{2}\sin{\left(\Delta_{w}+\theta \right)}^{2}\beta^{2}\left(\sigma_{w,y}^{2}+A^{2}\sin{\left(\Delta_{w}+\theta \right)}^{2}\beta^{2}\right)}{2\sigma_{w,y}^{4}}\\ & +\frac{A^{2}\cos{\left(\Delta_{w}+\theta \right)}^{2}\beta^{2}\left(\sigma_{w,x}^{2}+A^{2}\cos{\left(\Delta_{w}+\theta \right)}^{2}\beta^{2}\right)}{2\sigma_{w,x}^{4}}+O\left(\beta^{5}\right), \end{array}$$

and the second term is given by

∫∫−∞∞Q1(x,y)ϕ2β4dxdy=−3A4cos(Δw+θ)4σw,x4β412σw,x8−3A4sin(Δw+θ)4σw,y4β412σw,y8(40)−A4sin(Δw+θ)2cos(Δw+θ)2σw,x2σw,y2β42σw,x4σw,y4+O(β5)=−A4cos(Δw+θ)4β44σw,x4−A4sin(Δw+θ)4β44σw,y4(41)−A4sin(Δw+θ)2cos(Δw+θ)2β42σw,x2σw,y2+O(β5).

Then, we have

(42) $$\begin{array}{c} D\left(Q_{1}∥Q_{0}\right)=\frac{\beta^{4}}{4}{\left(\frac{A^{2}\cos{\left(\Delta_{w}+\theta \right)}^{2}}{\sigma_{w,x}^{2}}+\frac{A^{2}\sin{\left(\Delta_{w}+\theta \right)}^{2}}{\sigma_{w,y}^{2}}\right)}^{2}+O\left(\beta^{5}\right). \end{array}$$

## 5. Design of Amplitude Gain and Phase Angle

In Section 4.1 and Section 4.2, we provide the derivation of mutual information and KL divergence in noisy asymmetric systems. Combining with (32), the optimization target mutual information in (17) can be expressed as

(43) $$\begin{array}{c} I\left(Z_{b}^{n};D^{n}\right)=\frac{n\beta^{2}}{2}\left(\frac{B^{2}{\cos(\Delta_{b}+\theta )}^{2}}{\sigma_{b,x}^{2}}+\frac{B^{2}{\sin(\Delta_{b}+\theta )}^{2}}{\sigma_{b,y}^{2}}\right). \end{array}$$

Combining with (42), the covertness constraint in (18) can be expressed as

(44) $$\begin{array}{c} \frac{n\beta^{4}}{4}{\left(\frac{A^{2}\cos{\left(\Delta_{w}+\theta \right)}^{2}}{\sigma_{w,x}^{2}}+\frac{A^{2}\sin{\left(\Delta_{w}+\theta \right)}^{2}}{\sigma_{w,y}^{2}}\right)}^{2}\leq \epsilon . \end{array}$$

Next, we define

(45) $$\begin{array}{cc} G(\theta ) & ≜\frac{A^{2}\cos{\left(\Delta_{w}+\theta \right)}^{2}}{\sigma_{w,x}^{2}}+\frac{A^{2}\sin{\left(\Delta_{w}+\theta \right)}^{2}}{\sigma_{w,y}^{2}}, \end{array}$$

(46) $$\begin{array}{cc} T(\theta ) & ≜\frac{B^{2}{\cos(\Delta_{b}+\theta )}^{2}}{\sigma_{b,x}^{2}}+\frac{B^{2}{\sin(\Delta_{b}+\theta )}^{2}}{\sigma_{b,y}^{2}}, \end{array}$$

and the problem P1 can be rewritten as

(47)P2:maxβ,θnβ22T(θ)(48)s.t.nβ4G(θ)24≤ϵ,(49)0<β,(50)0<θ<π.

Considering the covertness constraint (48), we can obtain the maximal amplitude gain as

(51) $$\begin{array}{c} \beta ={\left(\frac{4\epsilon }{nG{\left(\theta \right)}^{2}}\right)}^{\frac{1}{4}}. \end{array}$$

Combining with (51) and (47), the mutual information can be expressed as

(52) $$\begin{array}{c} I\left(Z_{b}^{n};D^{n}\right)=\frac{\sqrt{n\epsilon }T\left(\theta \right)}{G(\theta )}. \end{array}$$

Then, the problem P2 can be rewritten as

(53)P3:maxθnϵT(θ)G(θ)(54)s.t.0<θ<π.

Evidently, within the specified interval for θ, the determination of the optimal angle θ and the maximization of mutual information can be resolved through a straightforward iterative algorithm. Concurrently, based on the optimal angle $\theta^{*}$, we can obtain the optimal amplitude gain as

(55) $$\begin{array}{c} \beta^{*}={\left(\frac{4\epsilon }{nG{\left(\theta^{*}\right)}^{2}}\right)}^{\frac{1}{4}}. \end{array}$$

Consequently, the original problem P1 has been fully resolved.

## 6. Numerical Results

In this section, we rigorously validate our proposed KL divergence and mutual information approximation methods across four distinct scenarios. With confirmed accuracy in these approximation techniques, we proceed to conduct a comprehensive performance analysis and optimal angle determination using our proposed approach. Ultimately, through a comparative performance evaluation between the optimal angle and alternative angles, we substantiate the efficacy and superiority of our introduced methodology.

**Figure 2:**
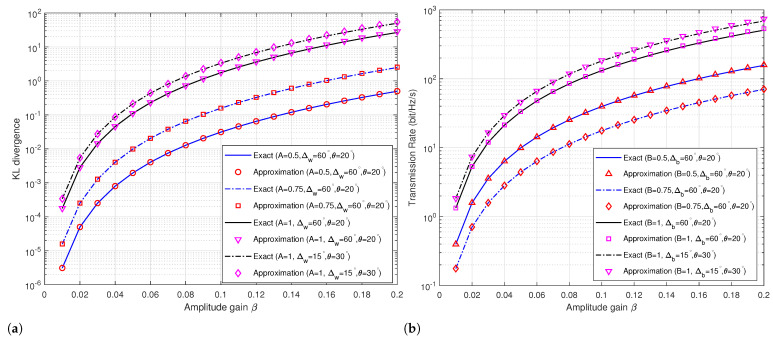
Performance analysis of covert communication. (**a**) KL divergence versus amplitude gain. (**b**) Transmission rate versus amplitude gain.

In Figure 2a,b, we configured four scenarios to assess the precision and Taylor expansion fitting accuracy under varying parameters. As illustrated in Figure 2a, the channel gain coefficient *A* at Willie’s location (*A* = 0.5, 0.75, and 1), initial phase angle Δ at 60 degrees and 15 degrees, and constellation modulation phase angle θ at 20 degrees and 30 degrees were varied. The gradual increase in KL divergence with the rise in amplitude gain β is evident. High fitting accuracy is consistently observed at both low and high amplitude instances, highlighting the precision of our employed methodology in delineating covertness performance in covert communication. In Figure 2b, corresponding to Bob’s location, the channel gain coefficient *B* is manipulated (0.5, 0.75, and 1), alongside the initial phase angle Δ and constellation modulation phase angle θ. Similar observations affirm the efficacy of our approach in characterizing transmission performance in covert communication at both low- and high-amplitude states.

**Figure 3:**
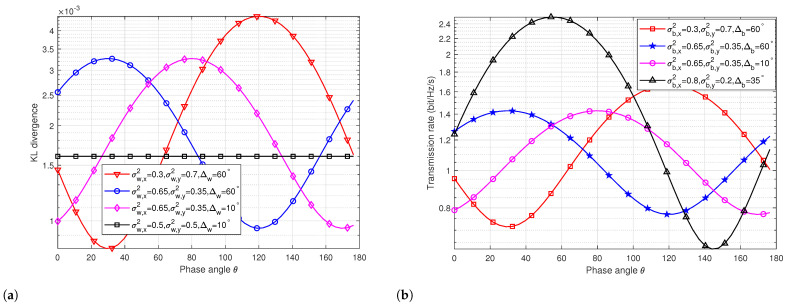
Performance analysis of covert communication in asymmetric noise system. (**a**) KL divergence versus phase angle. (**b**) Transmission rate versus phase angle.

Figure 3a,b illustrate the results of KL divergence and mutual information under scenarios of asymmetric channel noise. As depicted in Figure 3a, when Willie’s channel noise is symmetric ($\sigma_{w,x}=\sigma_{w,y}$), changes in the transmission angle do not impact KL divergence results, aligning with prior findings [11]. In Figure 3a, under conditions of asymmetric channel noise, optimal covertness, as represented by the optimal θ angle, varies based on the initial angle $\Delta_{w}$ and different *x*, *y*-axis channel noise, contingent on $\sigma_{w,x}$, $\sigma_{w,y}$, and $\Delta_{w}$. In Figure 3b, under asymmetric noise conditions, the optimal phase angle θ depends on the values of $\sigma_{b,x}$, $\sigma_{b,y}$, and $\Delta_{b}$ as the initial angle and *x*, *y*-axis channel noise change.

**Figure 4:**
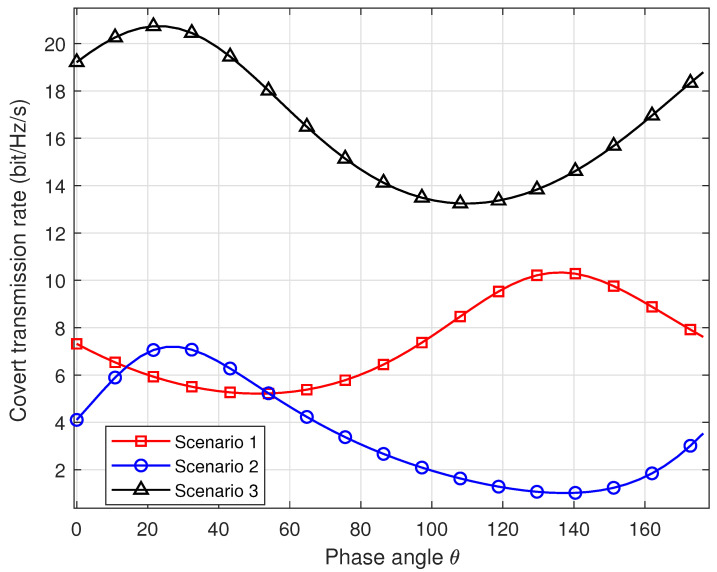
Covert transmission rate versus phase angle in three scenarios.

Figure 4 showcases the covert transmission rates versus the phase angle in three scenarios, aligning with the scenarios presented in Figure 3a,b. Specifically, in Scenario 1, $\sigma_{w,x}=0.3$, $\sigma_{w,y}=0.7$, $\Delta_{w}=10$ degrees, $\sigma_{b,x}=0.4$, $\sigma_{b,y}=0.5$, and $\Delta_{b}=60$ degrees; in Scenario 2, $\sigma_{w,x}=0.65$, $\sigma_{w,y}=0.35$, $\Delta_{w}=10$ degrees, $\sigma_{b,x}=0.55$, $\sigma_{b,y}=0.45$, and $\Delta_{b}=60$ degrees; in Scenario 3, $\sigma_{w,x}=0.65$, $\sigma_{w,y}=0.45$, $\Delta_{w}=10$ degrees, $\sigma_{b,x}=0.55$, $\sigma_{b,y}=0.35$, and $\Delta_{b}=30$ degrees. Our proposed methodology yields optimal θ angles of 135 degrees, 28 degrees, and 23 degrees for Scenarios 1, 2, and 3, respectively. Subsequent simulations will further validate these algorithmic results.

**Figure 5:**
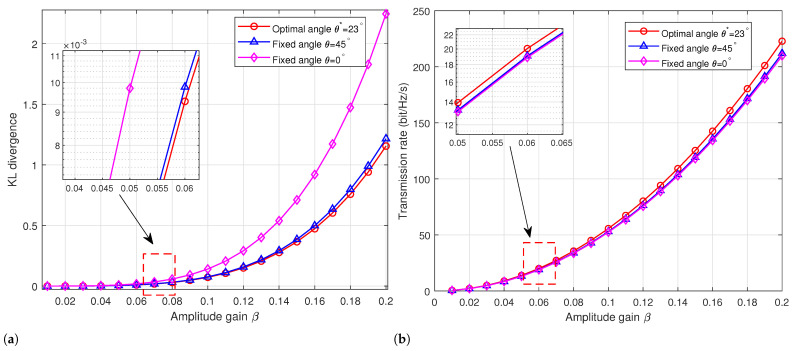
Performance comparison of difference phase angles. (**a**) KL divergence versus amplitude gain. (**b**) Transmission rate versus amplitude gain.

In Figure 5a,b, algorithmic results for Scenario 3 are validated. In order to contrast the performance of angle optimization using our proposed method ($\theta^{*}=23$), we employed fixed angles which have been utilized in previous works. Specifically, we considered the fixed angles denoted as θ=45 and θ=0 in papers [10,11], respectively, for comparative analysis. This approach ensures a rigorous comparison by utilizing established fixed-angle benchmarks from the literature as a baseline against which the efficacy of our angle optimization method can be evaluated.

As illustrated in Figure 5a, the optimal angle $\theta^{*}$ acquired through our algorithm not only attains superior covertness, as evidenced by lower KL divergence, but also achieves this at an equivalent amplitude gain β. This observation underscores the heightened efficiency of our algorithm in minimizing information divergence and enhancing the covertness aspect of the transmitted signal. Furthermore, in Figure 5b, the optimal angle $\theta^{*}$ is demonstrated to yield a higher transmission rate when compared to counterparts operating at the same amplitude gain β. This outcome highlights the ability of our proposed algorithm not only to improve covertness but also to enhance the overall transmission efficiency, a crucial aspect in covert communication scenarios. These compelling findings across both covertness and transmission rate metrics validate the efficacy and superiority of our proposed algorithm. The results contribute substantively to the understanding of angle optimization techniques in the context of communication systems, providing valuable insights for advancing the field of covert communication.

**Figure 6:**
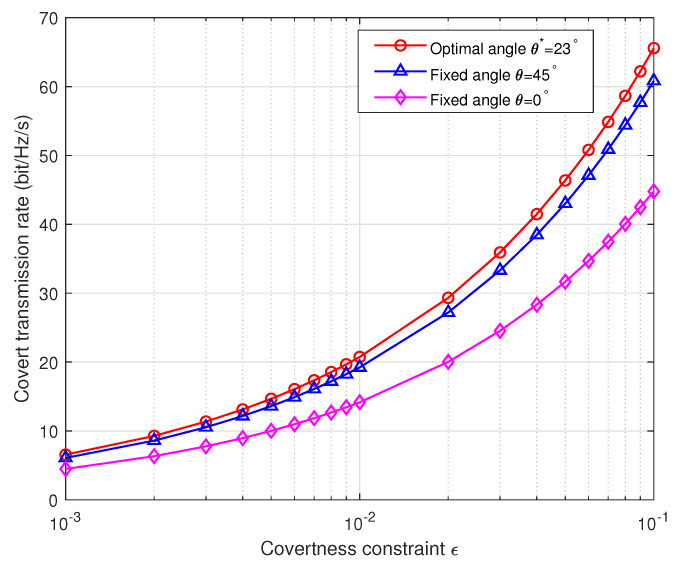
Covert transmission rate versus covertness constraint.

In Figure 6, we compare transmission rates under various covertness constraints ϵ based on Scenario 3, employing angles $\theta^{*}=23$ degrees, θ=45 degrees, and θ=0 degrees. The results indicate a substantial increase in transmission rates with higher ϵ values. Notably, adopting the optimal transmission angle $\theta^{*}=23$ degrees consistently leads to superior covertness transmission rates under equivalent covertness constraints. This observation underscores the efficacy and superiority of our proposed algorithm, which facilitates the determination of a globally optimal θ angle.

## 7. Conclusions

In this work, we investigated wireless covert communication in a multi-sensor asymmetric noise scenario. Specifically, we assessed the viability of wireless covert communication under the influence of asymmetric noise, which arises due to variations in signal frequencies and uneven sensor dispersion. KL divergence was employed as a metric to quantify the degree of covertness in communication, while mutual information served as a performance indicator for transmission. Utilizing these communication metrics, we optimized the transmission amplitude gain and signal phase angle in scenarios characterized by asymmetric noise. The key finding demonstrates that, in the presence of asymmetric noise, the selection of the phase angle plays a pivotal role in determining the trade−off between transmission rate and covert communication metrics. Additionally, we proposed a method to derive the optimal transmission amplitude and signal phase angle within the specified scenario. The numerical results substantiate the effectiveness and superiority of our proposed method, demonstrating its capability to maximize covert transmission rates.

## Data Availability

Data are contained within the article.

## Abbreviations

The following abbreviations are used in this manuscript:

WIFI	Wireless Fidelity
OFDM	Orthogonal frequency-division multiplexing
BPSK	Binary Phase Shift Keying
AWGN	Additive White Gaussian Noise
QPSK	Quadrature Phase Shift Keying
KL	Kullback–Leibler
IoT	Internet of Things
LDPC	Low Density Parity Check
